# Machine Learning Guided Design of Single–Phase Hybrid Lead Halide White Phosphors

**DOI:** 10.1002/advs.202101407

**Published:** 2021-07-14

**Authors:** Hailong Yuan, Luyuan Qi, Michael Paris, Fei Chen, Qiang Shen, Eric Faulques, Florian Massuyeau, Romain Gautier

**Affiliations:** ^1^ State Key Lab of Advanced Technology for Materials Synthesis and Processing Wuhan University of Technology Wuhan 430070 China; ^2^ Certara 54 Rue de Londres Paris 75008 France; ^3^ Université de Nantes, CNRS, Institut des Matériaux Jean Rouxel, IMN Nantes F‐44000 France

**Keywords:** high color rendering, machine‐learning, single‐phase white phosphors, tunable color temperature

## Abstract

Designing new single‐phase white phosphors for solid‐state lighting is a challenging trial–error process as it requires to navigate in a multidimensional space (composition of the host matrix/dopants, experimental conditions, etc.). Thus, no single‐phase white phosphor has ever been reported to exhibit both a high color rendering index (CRI ‐ degree to which objects appear natural under the white illumination) and a tunable correlated color temperature (CCT). In this article, a novel strategy consisting in iterating syntheses, characterizations, and machine learning (ML) models to design such white phosphors is demonstrated. With the guidance of ML models, a series of luminescent hybrid lead halides with ultra‐high color rendering (above 92) mimicking the light of the sunrise/sunset (CCT = 3200 K), morning/afternoon (CCT = 4200 K), midday (CCT = 5500 K), full sun (CCT = 6500K), as well as an overcast sky (CCT = 7000 K) are precisely designed.

## Graphical abstract

Through the guidance of machine learning tools, the host composition and dopants concentration of hybrid lead halides can be finely controlled to design a series of single‐phase white phosphors with ultra‐high color rendering (above 92) mimicking the light of the sunrise/sunset, morning/afternoon, midday, full sun, as well as an overcast sky.

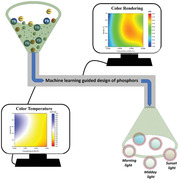

## 1 Introduction

In lighting and display industries, phosphor converted white light‐emitting diodes (pc‐wLEDs) have been widely adopted owing to their high luminous flux, high efficacy, and long lifespan.^[^ ^1^, ^2^ ^]^ Current pc‐wLEDs devices consist in a blue chip coated with yellow and red phosphors or an ultraviolet (UV) chip with red, green, and blue phosphors.^[^ ^3^ ^]^ The combination of emissions originating from the chip and the mixture of different phosphors provides an illumination over the entire visible spectrum.^[^ ^4^ ^]^ However, issues such as the reabsorption of emissions, the heterogeneous particle sizes, and the non‐uniformity of the luminescence properties from different phosphors make the white emission difficult to control and to be stabilized over time.^[^ ^5^, ^6^, ^7^ ^]^ As a result, low color rendering index (CRI) (typically below 80) is the main limitation of the commercialized wLEDs.^[^ ^8^, ^9^, ^10^ ^]^ In this context, the strategy of combining UV chips and a single‐phase white phosphor exhibiting multiple emission bands has recently drawn new attention. To design such phosphors, the intensity/wavelength of the emissions must be tuned. This control remains challenging because different emissions originating from a single‐phase material are commonly interdependent due to energy transfers. In addition, to reach high CRIs, the emission spectrum of the phosphor must be similar to the black body radiation but such spectrum changes significantly with the temperature. For these reasons, achieving tunable correlated color temperature (CCT) with ultra‐high CRI is very challenging. The previously reported white phosphors exhibited either a high CRI with a CCT which is not appropriate for solid‐state lighting, or an appropriate CCT with a poor CRI.^[^ ^8^, ^11^, ^12^, ^13^ ^]^ Thus, a methodology to precisely design white emitters with ultra‐high CRI and tunable CCT is essential for the concept UV chip + single‐phase white phosphor to be used in future pc‐wLED technology.

**Figure 1:**
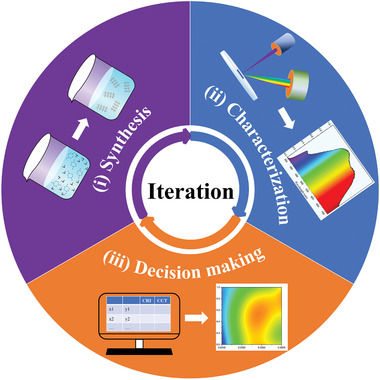
Schematic depiction of the discovery of new materials. Three steps: i) synthesis, ii) characterization, and iii) decision making are iterated.

Traditionally, the optimal experimental conditions for the synthesis of a material are achieved by multiple iterations of the three following steps: (i) Synthesis, (ii) Characterization, and (iii) Decision making (**Figure** **1**). After synthesis of a material, its properties are characterized, and analyzed to decide the next synthesis conditions. The 'decision making' step plays an important role in such iterative process. This step is entirely based on the scientists’ ability to rationalize the investigated chemical system which grows with the amount of experiments/results (both positive and negative results).^[^ ^14^ ^]^ However, the sophisticated mechanism hidden inside a multi‐dimensional space may prevent the scientists from deriving the optimal experimental conditions. For this reason, the optimization of properties is a time‐consuming and complex trial‐and‐error process. Machine‐learning (ML) models have been shown to be efficient solutions for such optimization problems. Although ML models have recently been used to predict the optical and piezoelectric properties of materials,^[^ ^14^, ^15^, ^16^ ^]^ the reported strategies do not typically incorporate the traditional approach of discovering new materials.^[^ ^17^, ^18^ ^]^ In this article, we proposed a novel strategy in which ML models substitute the human scientist decisions in step (iii) of the traditional iterative approach (Figure 1).

## 2 Results and Discussions

**Figure 2:**
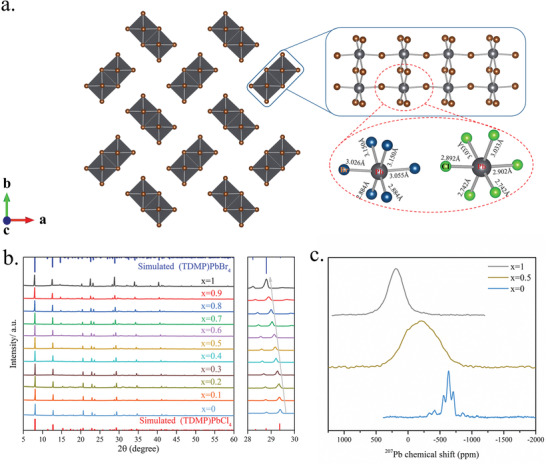
Synthesis and structural characterization of the (TDMP)Pb*X*_4_(*X* = Cl, Br) series. a) Representation of the crystal structure of (TDMP)Pb*X*_4_(*X* = Cl, Br) (the organic moieties are disordered between the lead halide chains); b) Powder X‐ray diffraction patterns of (TDMP)Pb(Br*_x_* Cl_1−_ *_x_*)_4_ (0 ≤ *x* ≤ 1); c)^207^Pb solid‐state NMR of (TDMP)Pb(Br*_x_* Cl_1−_ *_x_*)_4_ (*x*=0, 0.5, 1).

To demonstrate the possible design of single‐phase white phosphors with ultra‐high CRI and tunable CCT using ML tools, the Mn doped solid‐solution (TDMP)Pb(Br*_x_* Cl_1−_ *_x_*)_4_:*y*Mn (0 ≤ *x* ≤ 1, *y* is nominal) (TDMP = trans‐2,5‐dimethylpiperazine) was investigated. (TDMP)PbCl_4_ and (TDMP)PbBr_4_ compounds were previously reported to exhibit the same crystal structure (space group *P*2_1_ *m*) (**Figure** **2**a).^[^ ^19^, ^20^ ^]^ (TDMP)Pb*X*_4_ (*X* = Cl or Br) shows a packing of chains built from dimers of edge‐sharing Pb*X*_6_ octahedral connected through their corners. Such hybrid metal halides can exhibit two emissions originating from the recombination of free excitons (FE) and the recombination of self‐trapped excitons (STE).^[^ ^11^, ^19^ ^]^ No photoemission from the organic molecule is expected in (TDMP)Pb*X*_4_ as the isostructural (TDMP)Sn*X*_4_ compounds showed only a very weak photoemission originating from the recombination of free excitons. In addition, active dopants, such as Mn, give rise to a third emission originating from d–d transitions of Mn^2+^ (^4^T_1_ to ^6^A_1_).^[^ ^11^, ^21^, ^22^ ^]^ These three emissions cover the full visible spectrum providing opportunities to design a high CRI white phosphor. Previous works demonstrated that the halogens influence the recombination of the excitons (i.e., free excitons vs self‐trapped excitons),^[^ ^23^ ^]^ while the concentration of Mn influences the energy transfer from FE to STE and Mn.^[^ ^19^, ^22^ ^]^ Thus, in this case, an optimization in a multi‐dimensional system (CCT = f(*x*, *y*) and CRI = g(*x*,*y*)) is required to potentially design a white phosphor with ultra‐high CRI and tunable CCT.

To initiate the ML guided discovery of white phosphors, 55 samples of (TDMP)Pb(Br*_x_* Cl_1−_ *_x_*)_4_:*y*Mn with various ratios of n_Br_/n_(Cl+Br)_ (*x*) and concentrations of Mn (*y*) were synthesized (Experimental Section; and Table S2, Supporting Information). XRD patterns (Figure 2b; Figure S1, Supporting Information) suggested the (TDMP)Pb(Br*_x_* Cl_1−_ *_x_*)_4_ (0 ≤ *x* ≤ 1) and (TDMP)Pb(Br*_x_* Cl_1−_ *_x_*)_4_:*y*Mn compounds to be pure single phases.^20,21^ With the increase of Br content, the XRD peaks shifted to lower angles due to the difference in halogen radii. In addition, the elemental analyses of each compound were performed by energy‐dispersive X‐ray spectroscopy (EDX) measurements (Table S1, Supporting Information) and showed a very good agreement with nominal stoichiometries. These results were further supported at a more local scale through solid state NMR spectra. The ^207^Pb isotropic chemical shift is governed by the halogen chemical composition forming the octahedron surrounding the lead atom. The ^207^Pb MAS NMR spectrum of the compound with a nominal *x* of 0.5 (Figure 2c) shows no signal of (TDMP)PbBr_4_ or (TDMP)PbCl_4_ phases. The signal only consists in a symmetric broad line covering the full range between the signals of the pure [PbCl_6_] (−613 ppm) and pure [PbBr_6_] (191 ppm) octahedra. Thus, this result strongly suggests that the sample is homogeneous and that the halogen atoms are randomly dispersed within the crystal structure. The same conclusion can be drawn from the analysis of the ^13^C and ^15^N MAS NMR spectra (Figure S2, Supporting Information). The ^207^Pb NMR line width reflects the distribution of angles and lengths of the Pb─*X* bonds (*X* = Br or Cl). The line broadening with heavier anions is usually observed as the distribution increases with the softness of the Pb─*X* bond. Mixing Br and Cl within a [PbX6] octahedron further increases the distribution of angles and bond lengths and, therefore, the ^207^Pb NMR line width. As the ^207^Pb isotropic chemical shift depends on the chemical composition of its halogen shell, the first moment of the ^207^Pb spectra can be used to determine the *n*_Br_/*n*_(Cl+Br)_ ratio. By comparing the first moments of (TDMP)PbBr_4_ (191 ppm) and (TDMP)PbCl_4_ (−613 ppm) to the one (−202 ppm) of the sample with a nominal *x* of 0.5, one can calculate *x* = 0.49 in perfect agreement with the value determined by EDX (Table S1, Supporting Information).

**Figure 3:**
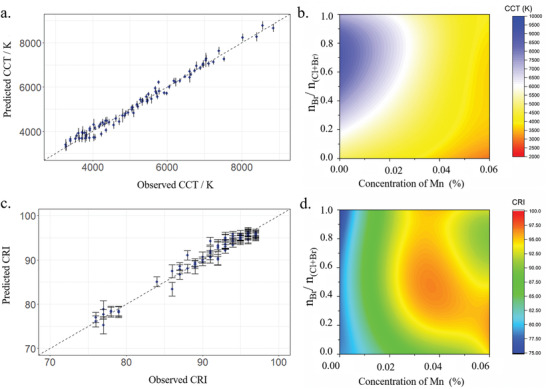
Model construction and validation for the final iteration. a) Comparison of predicted CCT versus observed CCT; b) Contour colormap of predicted CCT based on the regression models of CCT = f(*x*,*y*); c) Comparison of predicted CRI versus observed CRI; d) Contour colormap of predicted CRI based on the regression models of CRI = g(*x*,*y*) for (TDMP)Pb(Br*_x_* Cl_1−_ *_x_*)_4_:*y*Mn (*x* = n_Br_/n_(Cl+Br)_, *y* is the Mn concentration).

The CRI and CCT values of the initial series of 55 samples were calculated from the emission spectra (Table S2, Supporting Information). From these data, one cannot simply derive the rules to design a new material with an ultra‐high CRI for a specific CCT, because the relationships between the experimental conditions and these characteristics are complex. Thus, we attempted to quantify the relationship between the targeted properties (i.e., CRI and CCT) and the experimental conditions (i.e., *x* and *y*) through polynomial regression models. Considering the limited number of initial data points, we first built two 3‐order polynomial regression models CCT = f(*x*,*y*) and CRI = g(*x*,*y*) (Experimental Section, **Figure** **3**a,c; Figure S3, Supporting Information).

**Figure 4:**
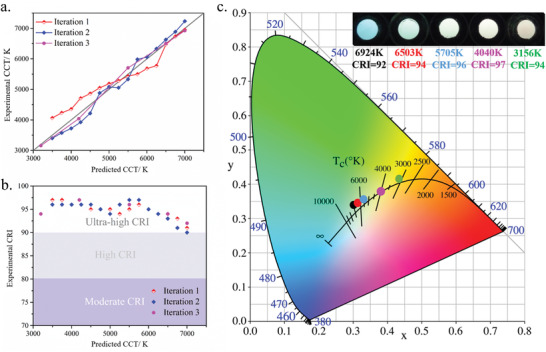
Performance of the predictive ML model. a) Comparison between predicted and experimental CCT for each iteration; b) Experimental CRI for different CCTs and for each iteration of the optimization cycle; c) CIE coordinates and photographies of the five final samples (obtained after iteration 3), with ultra‐high CRIs for a wide range of CCTs (≈3200–7000 K).

After building the models using the data from the initial series of 55 samples, the iteration between the three steps ((i) Synthesis, (ii) Characterization, and (iii) Decision making) was initiated to improve the performance of the decision‐making algorithm and optimize the properties of the phosphors. For this purpose, in each iterative cycle, 15 values of CCT were selected from the range 3500–7000 K. Using the model CCT = f(*x*,*y*), a set of (*x*,*y*) was derived for each selected CCT. And, for each (*x*,*y*) in this set, the CRI was calculated from the model CRI = g(*x*,*y*). Thus, for a specific CCT, the (*x*,*y*) with the optimal value of CRI could be determined. These optimal experimental conditions recommended by the regression models were then used for the next series of materials. After synthesis (i) and characterization (ii) of this new series of materials, additional experimental results ((*x*,y), CCT, CRI) could be obtained. These additional data were then added into the modeling dataset to refine the two models and make a new decision (iii) on the next experiments. The steps of the cycle (i)–(ii)–(iii) could then be reiterated. Through new iterations, the refinements of the two models resulted in an obvious increase of the prediction accuracy for CCT (**Figure** **4**a). In parallel, the CRI values tend to increase while the difference between experimental and predicted CRI decreased (Tables S3–S5, Supporting Information; Figure 4b). After two iterations, a total of 85 samples were obtained, allowing an upgrade of the regression models to 4‐order polynomials after model selection. Compared to the initial models, MAE (mean absolute error) decreased by 15.4% for the CCT model and 23.3% for the CRI model; RMSE (root mean squared error) decreased by 19.1% for the CCT model and 12.6% for the CRI model (Table S6, Supporting Information). In the 4‐order polynomials models, the CCTs are in the range 2000–10 000K, while the CRIs are in the range 75–100. Thus, owing to satisfactory accuracy and precision in the predictions (Figure 3b,d), these models were considered for a final iteration. In this final iteration, five different CCTs corresponding to the sunrise/sunset (CCT = 3200 K), morning/afternoon (CCT = 4200K), midday (CCT = 5500 K), full sun (CCT = 6500K) as well as overcast sky (CCT = 7000 K) were selected. The corresponding samples were synthesized and characterized (Figure S4 and Table S5, Supporting Information, and Figure 4c) and all five samples exhibited ultra‐high CRIs (above 92, the highest reaching 97). Thus, these compounds were the first reported series of single‐phase white phosphors with ultra‐high CRI for a broad range of CCTs. The ML approach allowed a 70‐fold reduction in the number of experiments needed relative to a grid‐based search. In addition, the photoluminescence quantum yield (PLQY) of these samples was above 28% (the highest PLQY is 43%) (Table S5, Supporting Information), which is higher than most of the reported single‐phase hybrid lead halides exhibiting white photoluminescence (including hybrid perovskites).^[^ ^24^, ^25^ ^]^ The differences in PLQYs of these five samples are due to the combination of different compositions in halogens influencing the trapping/detrapping of the excitons and different concentrations of Mn dopants which provide an efficient radiative pathway of the excitons.

**Figure 5:**
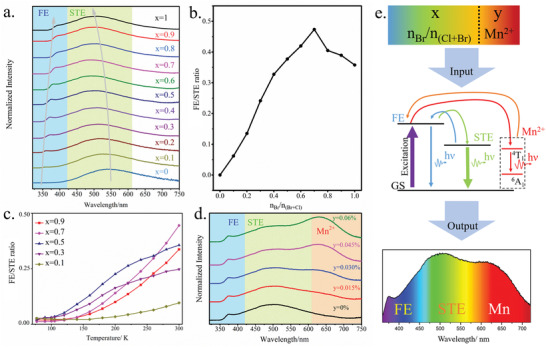
Optical properties and mechanisms of photoluminescence. a) Emission spectra of (TDMP)Pb(Br*_x_* Cl_1−_ *_x_*)_4_ (0 ≤ *x* ≤ 1); b) Evolution of the FE/STE ratio with n_Br_/n_(Cl+Br)_ at room temperature for (TDMP)Pb(Br*_x_* Cl_1−_ *_x_*)_4_ (0 ≤ *x* ≤ 1); c) Evolution of the FE/STE ratio with temperature for (TDMP)Pb(Br*_x_* Cl_1−_ *_x_*)_4_ (0 ≤ *x* ≤ 1); d) Emission spectra of (TDMP)Pb(Br_0.5_Cl_0.5_)_4_:Mn with different concentrations of Mn^2+^ and e) Mechanism of the photoluminescence for (TDMP)Pb(Br*_x_* Cl_1−_ *_x_*)_4_:*y*Mn (0 ≤ *x* ≤ 1).

The mechanisms responsible for the evolutions of the CCT and CRI with (*x*,y) can be further investigated through the analysis of the optical properties of the (TDMP)Pb(Br*_x_* Cl_1−_ *_x_*)_4_:*y*Mn (0 ≤ *x* ≤ 1) solid‐solution (**Figure** **5**a; and Figure S5, Supporting Information). The emission spectra of the Mn doped lead halide materials are composed of emissions originating from the recombination of FE (blue region), the recombination of STE (green/yellow region), and d–d transitions of Mn (red region). Thus, the relative intensity and wavelengths of FE, STE, and Mn emissions directly influence the CCT and CRI. To better rationalize these mechanisms, the effects of Mn doping were decoupled from the effect of the recombination of FE versus STE by analyzing separately the non‐doped compounds and the manganese doped compounds.

For non‐doped compounds (i.e., *y* = 0), the CCT of most samples (all samples with *x* > 0.2) are above 6000 K (Figure 3b) and the CRI of all the samples is below 80 (Figure 3d; PLQYs in Table S7, Supporting Information) due to the absence of the red emission from Mn dopants. For these non‐doped compounds with *x* < 0.7, the FE/STE ratio increases with *x* (contribution of blue emission increases relatively to the green/yellow emission) (Figure 5b), leading to higher CCTs despite a blue shift of the STE emission. For *x* = 0.7, the ratio FE/STE reaches its maximum and, consequently, the CCT reaches the highest value (9367 K). For compounds with *x* > 0.7, the FE/STE ratio decreases with *x* (the contribution of blue emission decreases relatively to green/yellow emission) (Figure 5b) and a red‐shift of STE emission is observed, leading to lower CCTs. These different evolutions of FE and STE emissions with *x* are related to the equilibrium between trapping / detrapping of the excitons. In (TDMP)PbCl_4_ (*x* = 0), the detrapping process is not thermally activated at room temperature due to the high detrapping barrier.^[^ ^19^ ^]^ Thus, no exchange between free and self‐trapped exciton states is observed. From (TDMP)PbCl_4_ (*x* = 0) to (TDMP)Pb(Br_0.7_Cl_0.3_)_4_ (*x* = 0.7), the detrapping barrier decreases and, consequently, the FE/STE ratio increases gradually. From (TDMP)Pb(Br_0.7_Cl_0.3_)_4_ (*x* = 0.7) to (TDMP)PbBr_4_ (*x* = 1), this FE/STE ratio should increase as the detrapping decreases. However, the ratio decreases suggesting that an additional parameter influences FE and STE. As the exchange between free and self‐trapped states is temperature dependent, the evolution of the ratio FE/STE with temperature was investigated for (TDMP)Pb(Br*_x_* Cl_1−_ *_x_*)_4_ (*x*=0.1, 0.3, 0.5, 0.7, 0.9) (Figure 5c; Figure S6, Supporting Information). The detrapping of self‐trapped excitons is thermally activated and leads to the increase of the FE/STE ratio with temperature for all samples. Figure S7, Supporting Information, represents the evolution of FE and STE emissions with temperature. When the temperature increases, the STE emission decreases, while FE emission increases first and then decreases. Such behavior of FE emission with temperature can be explained by two opposite phenomena: (i) the detrapping which is thermally activated and leads to the increase of the FE emission with temperature, and (ii) the non‐radiative recombination of the excitons leading to the decrease of FE emission with temperature. Thus, the FE emission increases at low temperature when phenomenon (i) is stronger than (ii) but decreases at high temperatures when phenomenon (i) is weaker than (ii). As these two phenomena are not only dependent on the temperature but also on the chemistry, the ratio FE/STE reaches a maximum at different *x* values for different temperatures (e.g., x = 0.5 at 200 K, x = 0.7 at room temperature). In addition, the FE and STE emissions show different shifts with *x*: the STE emission blue‐shifts from 530 nm (for *x* = 0) to 503 nm (for *x* = 1), which is related with the effectiveness of hybridization and the exciton binding energy,^[^ ^26^ ^]^ while the FE emission shows a red‐shift as heavier halogens lead to narrower band gaps.^[^ ^27^, ^28^ ^]^

For doped compounds (i.e., *y* > 0), the CCTs cover a broad range (≈2000–10 000 K) and the CRI can increase significantly (the highest value reaching 97). The series of samples (TDMP)Pb(Br*_x_* Cl_1−_ *_x_*)_4_:*y*Mn (*x* = 0.5, 0 ≤ *y* ≤ 0.06%), (Figure 5d; see Figure S8, Supporting Information, for materials with other values of *x*), was selected to investigate the influence of Mn concentration on CCT and CRI values. The similar excitation spectra of STE and Mn emissions (Figure S9, Supporting Information), suggest that both emissions originate from the FE states. Thus, the relative intensities of the three emissions are not influenced by the excitation wavelength which is an advantage for solid‐state lighting applications.^[^ ^21^, ^22^ ^]^ With the increasing concentration of Mn^2+^, the intensity of the red emission will increase leading to the decrease of the CCT. In parallel, such increase of red emission with *y*, leads to an increase of the CRI from *y* = 0 % to *y* = 0.04 % and decrease from *y* = 0.04 % to *y* = 0.06 % because of an excess of the red emission relatively to blue/green emissions (Figure 5d). For the other series of compounds (*x* ≠ 0.5), the highest CRI values are reached for different *y* values as the contribution from blue emission (FE) and green/yellow emission (STE) should be balanced with the red emission (Mn^2+^) to obtain an optimal CRI. Such phenomenon illustrates the difficulty of predicting optimal CRI without using ML tools in step (iii). Thus, a scientist would have to navigate through a multi‐dimensional system with nonlinear relationships between variables. The mechanism describing how (*x*,*y*) influences the FE, STE, and Mn emissions (and consequently, the CCT and CRI values) is summarized in Figure 5e. Through controlling the ratio *n*_Br_/*n*_(Cl+Br)_, the band gap is tuned influencing the intensity and wavelength of FE emission (blue) and STE emission (green/yellow). Through controlling the concentration of Mn, the red emission can also be tuned.

## 3 Conclusion

In summary, tuning the different spectral contributions of (TDMP)Pb(Br*_x_* Cl_1−_ *_x_*)_4_:*y*Mn (0 ≤ *x* ≤ 1) through the ratio *x* = *n*_Br_/*n*_(Cl+Br)_ and the concentration of *y* = Mn^2+^, to obtain a white emission with specific CCT and ultra‐high CRI is challenging. Due to the complex relationships between FE, STE, and Mn emissions as well as the red shift of FE emission and blue shift of STE emission with *x*, the CCT and CRI are unpredictable without the intervention of ML tools. Based on 85 samples and an iteration process, regression models CCT=f(*x*,*y*) and CRI=g(*x*,*y*) were built and refined. Such ML‐guided discovery through a fully automated decision making process enabled us to design single‐phase white phosphors with an ultra‐high CRI (above 92) and a given CCT (from 3200 to 7000K). It is also important to note that this ML approach can be directly used (i.e., with the same code) for any hybrid metal halides in which the composition of halogens and the concentration of dopants (Mn^2+^, Eu^2+^, Ce^3+^, etc.) can be tuned. This work not only highlights the potential of hybrid metal halides for pc‐wLEDs, but also shows that ML based strategies in which the experimental work and modeling are iterated can be very powerful at guiding the synthesis of multifunctional materials. We believe this iteration, which is very often overlooked in previous studies where new materials are theoretically predicted, is primordial for the ML models to make accurate predictions and optimal decisions.

## 4 Experimental Section

### Materials Synthesis

(TDMP)Pb(Br*_x_* Cl_1−_ *_x_*)_4_ (0 ≤ *x* ≤ 1) samples were synthesized from a mixture of 0.004 × *x* mol PbBr_2_ (Alfa Aesar, 99.95%), 0.004 × (1−*x*) mol PbCl_2_ (Alfa Aesar, 99.95%) and 0.005 mol trans‐2,5‐dimethylpiperazine (alfa Aesar, 98%) added into 5 × *x* mL HBr (Alfa Aesar, 48%), 5 × (1−*x*) mL HCl (Alfa Aesar, 37%) and 5 ml H_2_O. After heating under reflux for 3 h with agitation, the solutions were cooled down and a white powder was recovered by filtration and washed with ethanol. (TDMP)Pb(Br*_x_* Cl_1−_ *_x_*)_4_:*y*Mn (0 ≤ y ≤ 0.06%) samples were synthesized from a mixture of 0.002 × *y* mol MnCl_2_ and 0.002 mol (TDMP)Pb(Br*_x_* Cl_1−_ *_x_*)_4_ by grinding and then heating at 150 °C for 5 h.

### Characterizations—Powder X‐Ray Diffraction (XRD)

Phase identification was performed on an X‐ray diffractometer (D8 Bruker) with Cu K*α* radiation. The patterns covered an angular range 5° < 2*θ* < 90 ° with 0.02° per step.

### Characterizations—Energy‐Dispersive X‐Ray spectroscopy (EDX)

The composition of samples was analyzed by the X‐ray detector device attached to the SEM microscope (JEOL 5800LV).

### Characterizations—Solid‐State NMR

^1^H‐^13^C and ^1^H‐^15^N CP‐MAS (cross polarization and magic angle spinning) spectra were acquired on a 500 MHz Bruker Avance III NMR spectrometer using a 4 mm MAS probe. The CP contact time was 3 ms and the recycle time was 3 s. MAS speed was set to 10 and 5 kHz for ^13^C and ^15^N spectra, respectively. ^207^Pb solid state NMR was performed at 302 K on a 300 MHz Bruker Avance III or NEO spectrometer using a 4 mm MAS probe. The ^207^Pb MAS NMR spectra were acquired with a rotor synchronized Hahn echo sequence (*π*/2–*τ*–*π*–*τ*–acq) with *τ* equal to one rotor period. The radio‐frequency field was 90 kHz. The recycle time was set to 2 s and the MAS speed to 14 kHz. Chemical shifts were referenced to Pb(CH_3_)_4_ for ^207^Pb, to TMS for ^13^C and to liquid NH_3_ for ^15^N.

### Characterizations—Photoluminescence

All measurements were carried out on a Horiba Jobin–Yvon Flurolog 3 equipped with a 450 W xenon lamp. Photoluminescence emission (PL) and excitation (PLE) spectra were acquired by means of R13456 PMT detector. The optimal excitation wavelength for each sample was selected to measure the emission spectra and calculate the CCT / CRI. Samples were placed in an Oxford cryostat for cooling down to 77K. PLQYs were quantified using the quanta‐phi accessory from Horiba on the Fluorolog (absolute quantum yield measurements). The CCT and CRI values were calculated from the emission spectra using a modified version of the program pspectro.^[^ ^29^ ^]^

### Characterizations—UV–Vis Spectroscopy

Optical reflection spectra were acquired using a Perkin lambda 1050 equipped with a 150 mm integrating sphere. Reflection spectra were transformed into absorption spectra with the Kubelka‐Munk equation: (1−*R*)^2^/2*R*, where *R* is the reflectance.

### Decision Making Models—Establishment and Selection of the Regression Models

The polynomial regression model was used to describe the non‐linear relationship between the descriptors and properties. A frequentist optimization was selected because the established model could efficiently provide the experimental conditions for any desired color temperature after the iterations are processed. The models were built for CCT and CRI using R package {caret}, R version 3.6.3.

Initially, 55 samples were available (Table S2, Supporting Information). Thus, the initial models did not go beyond 3‐order polynomial to balance the data versus the number of parameters to be estimated (Note that attention was paid to not have less than five data (i.e., samples) per parameter in the authors' modeling practice). The authors built 1, 2, and 3‐order polynomial regression models by repeating 50 times the 10 folder cross validation (for CCT and CRI respectively). After comparing the root mean square error (RMSE), mean absolute error (MAE), and R‐Squared, it was decided to initialize the iteration process with the 3‐order polynomial regression models for both CCT and CRI models where the lowest RMSE and MAE and the highest R‐Squared were observed (Figures S10A,B, Supporting Information). After building the initial models, they were used to guide 15 new experiments, which were expected to provide phosphors with the expected CCTs and optimal CRIs. Here, 15 CCTs were selected every 250 K between 3500 to 7000 K (Table S3, Supporting Information).

After the first iteration, 15 new samples were added to the authors' dataset (70 samples in total). Considering the sample size limit, it was still not possible to go beyond 3‐order polynomials. After comparing the RMSE, MAE, and R‐Squared of the 1, 2, and 3‐order polynomial regression models for CCT and CRI, respectively, the 3‐order polynomial models were selected to update the initial CCT and CRI models (Figure S10C,D, Supporting Information). The updated models were used to guide the 15 new experiments (Table S4, Supporting Information).

After the second iteration, another 15 samples were added to the authors' dataset (85 samples in total). Such sample size allowed the authors to test 4‐order polynomial. After comparing the RMSE, MAE, and R‐Squared of the 1, 2, 3, and 4‐order polynomial regression models for CCT and CRI, the authors finally selected the 4‐order polynomial regression models for both CCT and CRI models (Figure S10E,F, Supporting Information).

### Decision Making Models—Decision Making

In each iteration step, the CCT and CRI models were used to predict the optimal experimental conditions of the phosphors with targeted CCT and optimal CRI. Thus, for each CCT_target, *x* ∈ {0, 0.01, 0.02, …, 1} and *y* ∈ [0, 0.00001, 0.00002, …0.0006]; the function CCT_target = ⨍(x, y) was solved using *R* package {stats} to obtain a set of (*x*,*y*). Then, for each (*x*,*y*) of this set, the CRI was predicted using the model CRI = g(*x*,*y*), and the (*x,y*) with the maximum CRI value was selected as the final prediction to guide the new experiment.

### Decision Making Models—R Code

Executable R codes (using the data of 85 samples) are provided in a separate file. All the calculation details are included in the R codes.

## Conflict of Interest

The authors declare no conflict of interest.

## Supporting information

Supporting Information

## Data Availability

Research data are not shared.
