# Supplementary material for: Machine Learning Guided Design of Single–Phase Hybrid Lead Halide White Phosphors
Source: Adv Sci (Weinh). 2021 Jul 14;8(19):2101407. doi: 10.1002/advs.202101407 (PMC8498859; doi:10.1002/advs.202101407)
Supplement: Supplementary file 1 — Supporting Information [file ADVS-8-2101407-s001.pdf]

© 2021 Wiley-VCH GmbH

ADVANCED SCIENCE
Open Access

Supporting Information

for *Adv. Sci.*, DOI: 10.1002/advs.202101407

Machine Learning Guided Design of Single–Phase Hybrid Lead Halide White Phosphors

*Hailong Yuan, Luyuan Qi, Michael Paris, Fei Chen, Qiang Shen, Eric Faulques, Florian Massuyeau, Romain Gautier**

Machine Learning Guided Design of Single–Phase Hybrid Lead Halide White Phosphors

*Hailong Yuan, Luyuan Qi, Michael Paris, Fei Chen, Qiang Shen, Eric Faulques, Florian Massuyeau, Romain Gautier**

Table S1. Energy-dispersive X-ray spectroscopy (EDX) results of (TDMP)Pb(Br_xCl_{1-x})$_4$ samples.

Nominal x	A% Cl	A% Br	A% Pb	Measured x
x=1	0	79.797	20.203	1
x=0.9	7.595	71.423	20.982	0.904
x=0.8	18.213	61.173	20.614	0.771
x=0.7	25.378	53.881	20.741	0.680
x=0.6	34.846	45.245	19.908	0.565
x=0.5	40.776	38.817	20.406	0.488
x=0.4	46.415	32.785	20.798	0.414
x=0.3	54.867	24.906	20.227	0.312
x=0.2	60.918	19.013	20.069	0.238
x=0.1	70.120	9.422	20.457	0.120
x=0	79.903	0	20.097	0

Table S2. Color Rendering Index (CRI), Correlated Color Temperature (CCT) of (TDMP)Pb(Br_xCl_{1-x})$_4$: yMn samples in screening process (55 samples) .

(TDMP)Pb(Br_xCl_{1-x})$_4$: yMn		CRI	CCT(K)	(TDMP)Pb(Br_xCl_{1-x})$_4$: yMn		CRI	CCT(K)
x=1	y=0%	77	7034	x=0.4	y=0%	79	7383
	y=0.015%	86	6597		y=0.015%	89	6179
	y=0.030%	95	5478		y=0.030%	95	5216
	y=0.045%	95	4270		y=0.045%	96	4066
	y=0.060%	92	3728		y=0.060%	95	3641
x=0.9	y=0%	76	8395	x=0.3	y=0%	79	6786
	y=0.015%	87	6970		y=0.015%	88	5776
	y=0.030%	96	5712		y=0.030%	93	4945
	y=0.045%	93	4385		y=0.045%	97	3924
	y=0.060%	91	3832		y=0.060%	96	3544
x=0.8	y=0%	76	8563	x=0.2	y=0%	79	6222
	y=0.015%	87	7040		y=0.015%	87	5330
	y=0.030%	97	5686		y=0.030%	92	4624
	y=0.045%	93	4346		y=0.045%	95	3791
	y=0.060%	92	3829		y=0.060%	96	3403
x=0.7	y=0%	77	9367	x=0.1	y=0%	79	5573
	y=0.015%	89	7526		y=0.015%	84	5101
	y=0.030%	97	5917		y=0.030%	88	4563
	y=0.045%	91	4441		y=0.045%	92	4024
	y=0.060%	90	3919		y=0.060%	96	3283
x=0.6	y=0%	78	8846	x=0	y=0%	77	4806
	y=0.015%	90	7116		y=0.015%	86	4033
	y=0.030%	97	5741		y=0.030%	89	3617
	y=0.045%	93	4308		y=0.045%	92	3277
	y=0.060%	91	3796		y=0.060%	93	3040
x=0.5	y=0%	78	8025				
	y=0.015%	89	6605				
	y=0.030%	97	5462				
	y=0.045%	94	4179				
	y=0.060%	93	3711				

Table S3. Predicted CCT/CRI and experimental CCT/CRI of $(TDMP)Pb(Br_xCl_{1-x})_4$: yMn samples (Iteration 1, 15 samples).

x ($n_{Br}/n_{(Cl+Br)}$)	y (Concentration of Mn)	Predicted CCT	Experimental CCT	Prediction CRI	Experimental CRI
0.28	0.00054	3500	4063	95.15	97
0.33	0.00050	3750	4234	95.17	97
0.37	0.00047	4000	4354	95.20	96
1	0.00044	4250	4712	95.22	96
1	0.00040	4500	4862	95.69	95
1	0.00036	4750	5050	95.75	95
1	0.00033	5000	5186	95.50	95
0.95	0.00032	5250	5285	95.05	94
0.86	0.00032	5500	5435	94.64	95
0.74	0.00031	5750	5498	94.26	96
0.73	0.00028	6000	5684	93.74	95
0.72	0.00025	6250	5772	93.09	94
0.71	0.00023	6500	6471	92.22	94
0.71	0.00020	6750	6736	91.23	93
0.7	0.00018	7000	6952	90.07	91

Table S4. Predicted CCT/CRI and experimental CCT/CRI of $(TDMP)Pb(Br_xCl_{1-x})_4$: yMn samples (Iteration 2, 15 samples).

x ($n_{Br}/n_{(Cl+Br)}$)	y (Concentration of Mn)	Predicted CCT	Experimental CCT	Prediction CRI	Experimental CRI
0.25	0.00057	3500	3398	95.39	96
0.31	0.00052	3750	3576	95.41	96
0.36	0.00048	4000	3722	95.49	96
0.41	0.00045	4250	3925	95.55	96
0.46	0.00043	4500	4215	95.58	96
1	0.00038	4750	4882	95.59	95
1	0.00034	5000	5080	95.55	94
0.63	0.00036	5250	5053	95.29	96
0.68	0.00033	5500	5332	95.02	97
0.7	0.00030	5750	5985	94.59	97
0.71	0.00027	6000	6059	93.99	95
0.7	0.00024	6250	6333	93.21	94
0.7	0.00022	6500	6630	92.27	93
0.7	0.00019	6750	6887	91.15	91
0.69	0.00016	7000	7234	89.85	90

Table S5. Predicted CCT/CRI and experimental CCT/CRI and photoluminescence quantum yield (PLQY) of $(TDMP)Pb(Br_xCl_{1-x})_4$: yMn samples (Iteration 3, 5 samples).

x ($n_{Br}/n_{(Cl+Br)}$)	y (concentration of Mn)	Predicted CCT	Experimental CCT	Predicted CRI	Experimental CRI	PLQY
0.06	0.00059	3200	3156	94.6	94	28%
0.38	0.00044	4200	4040	96.2	97	39%
0.59	0.00030	5500	5705	96.1	96	43%
0.71	0.00022	6500	6503	93.2	94	38%
0.72	0.00018	7000	6924	90.7	92	40%

Table S6. The RMSE (Root Mean Square Error), MAE (mean absolute error) and R-Squared of the selected CCT and CRI models in the iteration process.

**CCT model**			
	RMSE	MAE	R-squared
Initial model	285.3018	218.8424	0.9798423
Iteration 1	283.1391	215.9855	0.9737001
Iteration 2	230.8572	185.0859	0.9786136
**CRI model**			
	RMSE	MAE	R-squared
Initial model	1.757575	1.454173	0.9289891
Iteration 1	1.644130	1.346596	0.9297672
Iteration 2	1.536169	1.1152176	0.9329980

Table S7. PLQYs of $(TDMP)Pb(Br_xCl_{1-x})_4$ samples.

Samples: $(TDMP)Pb(Br_xCl_{1-x})_4$ (nominal values)	PLQY
x=1	45%
x=0.9	54%
x=0.8	52%
x=0.7	51%
x=0.6	49%
x=0.5	45%
x=0.4	43%
x=0.3	41%
x=0.2	32%
x=0.1	30%
x=0	9%

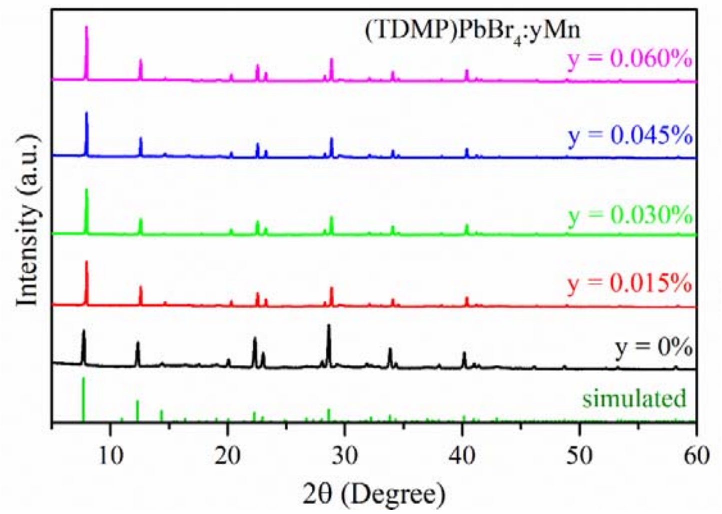

Figure S1. Powder XRD patterns of (TDMP)PbBr$_4$:yMn.

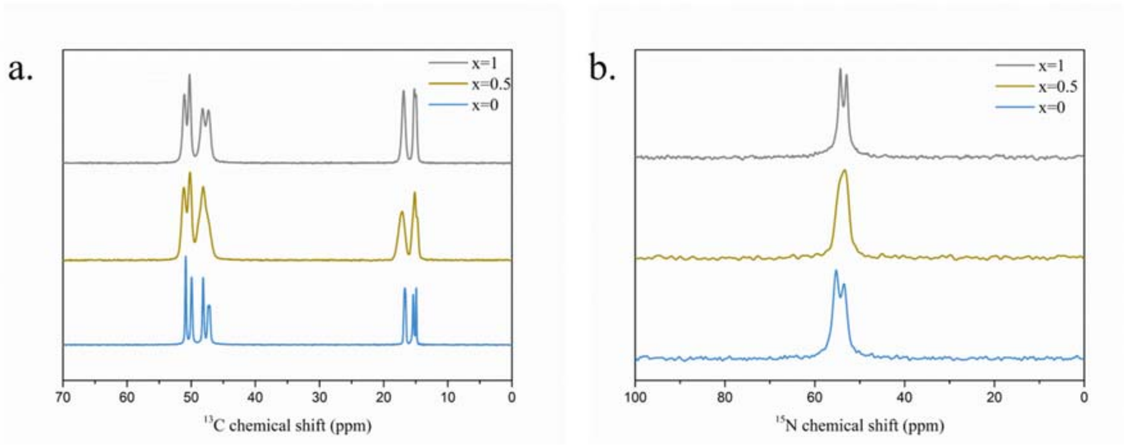

Figure S2. a. ^{1}H-^{13}C and b. ^{1}H-^{15}N CP-MAS NMR of (TDMP)Pb(Br_xCl_{1-x})$_4$ (x = 0, 0.5, 1).

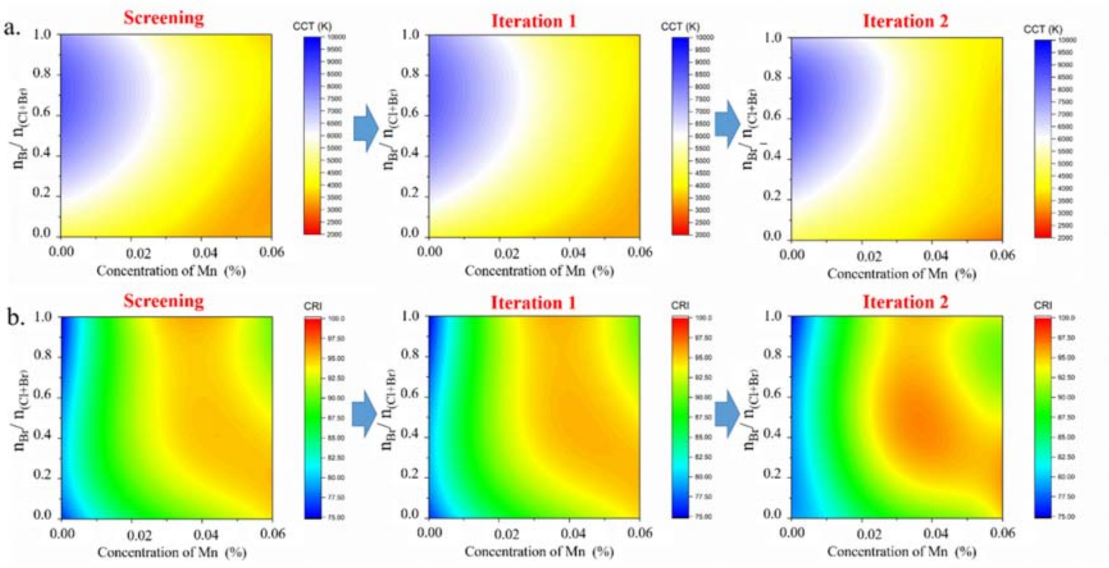

Figure S3. Evolution of the regression models after building/iteration a. CCT=ƒ(x, y) and b. CRI=ƒ(x, y), x= $n_{Br}/n_{(Cl+Br)}$, y is concentration of Mn. (Screening: 55 data points, Iteration 1: 55 data points + 15 new experiments, Iteration 2: 55 data points + 15 experiments+ 15 new experiments).

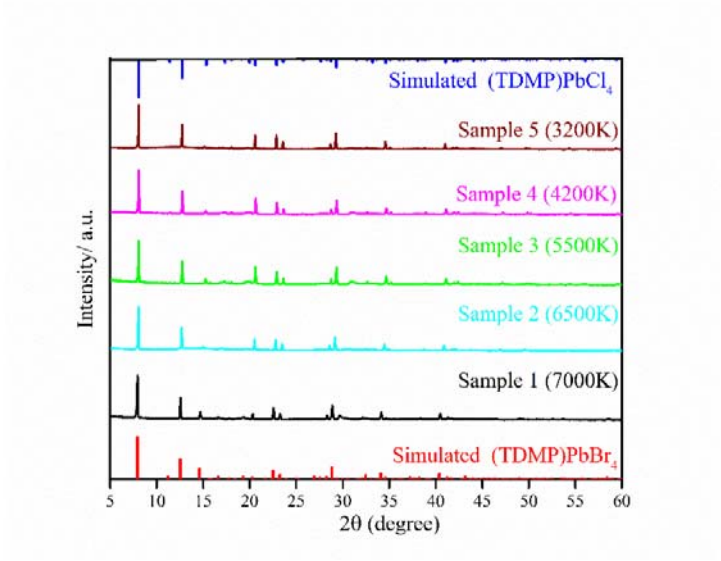

Figure S4. Powder XRD patterns of five selected samples (Iteration 3, Table S5) with different CCTs.

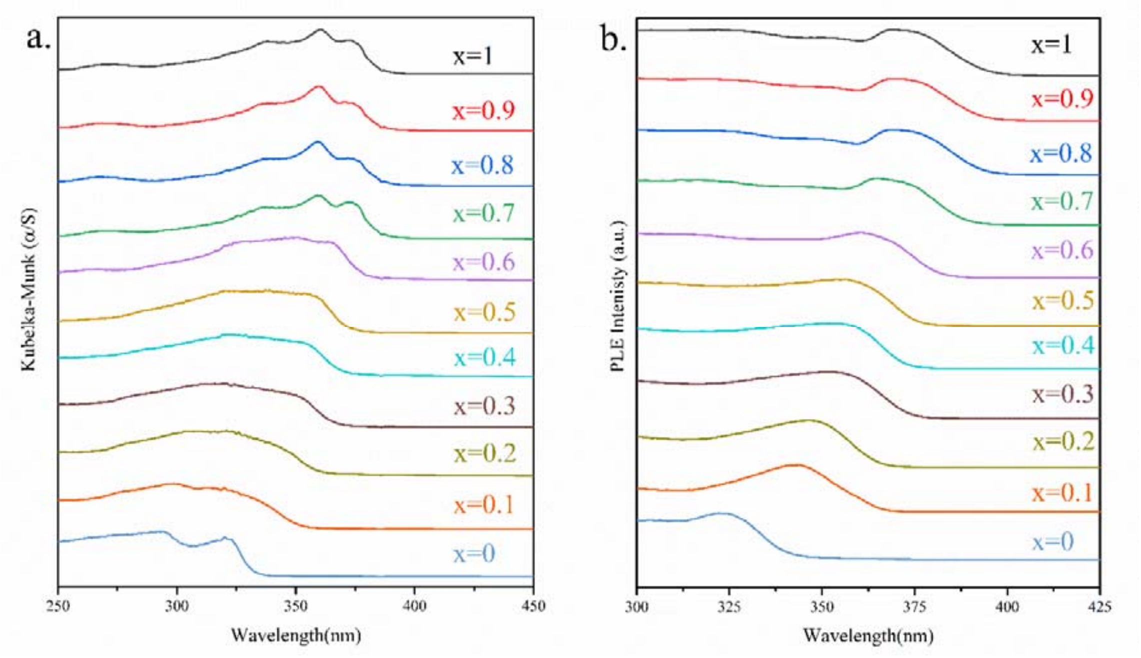

Figure S5. Optical properties of (TDMP)Pb(Br_xCl_{1-x})$_4$ (0≤x≤1) a. UV-visible diffuse reflectance spectra and b. excitation spectra.

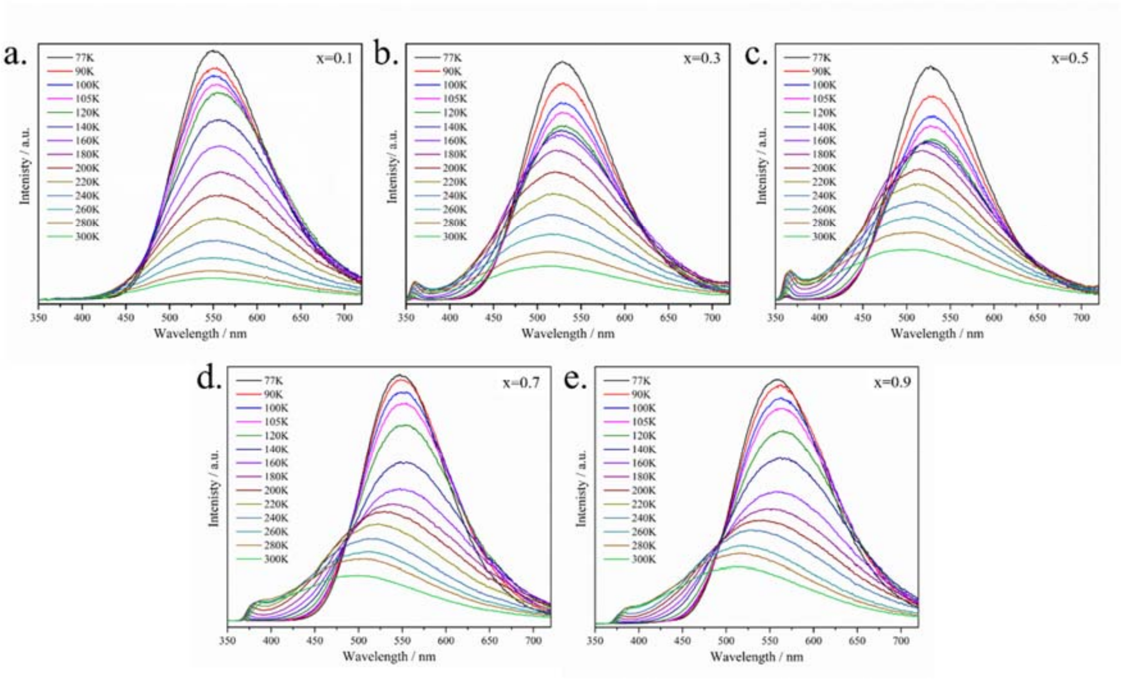

Figure S6. Evolution of PL emissions with temperature for (TDMP)Pb(Br_xCl_{1-x})$_4$ compounds. a. x=0.1; b. x=0.3; c. x=0.5; d. x=0.7; e. x=0.9.

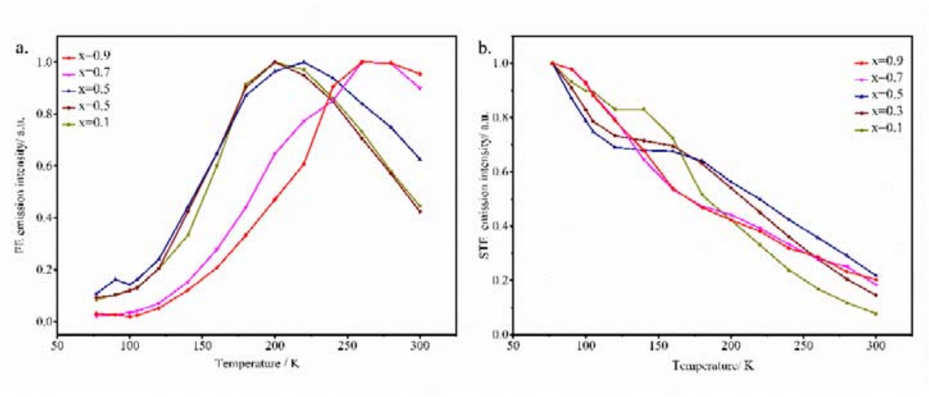

Figure S7. Evolution of FE and STE emissions with temperature for (TDMP)Pb(Br_xCl_{1-x})$_4$ compounds. a. FE; b.STE.

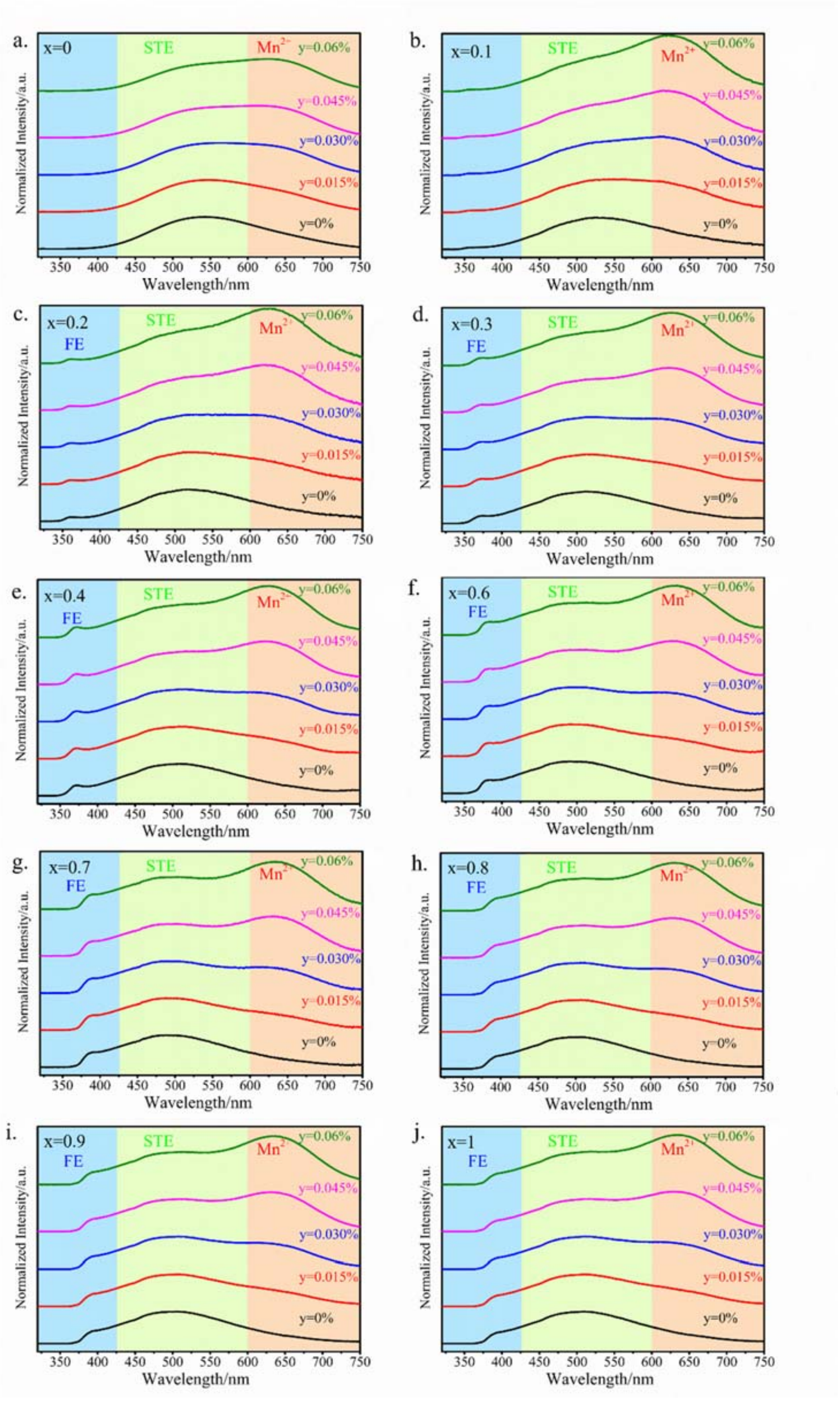

Figure S8. Optical properties of Mn^{2+} doped (TDMP)Pb(Br_xCl_{1-x})$_4$. Emission spectra for different ratio x= $n_{Br}/n_{(Cl+Br)}$ and concentration of $Mn^{2+)}$. a. x=0; b. x=0.1; c. x=0.2; d. x=0.3; e. x=0.4; f. x=0.6; g. x=0.7; h. x=0.8; i. x=0.9; j. x=1.

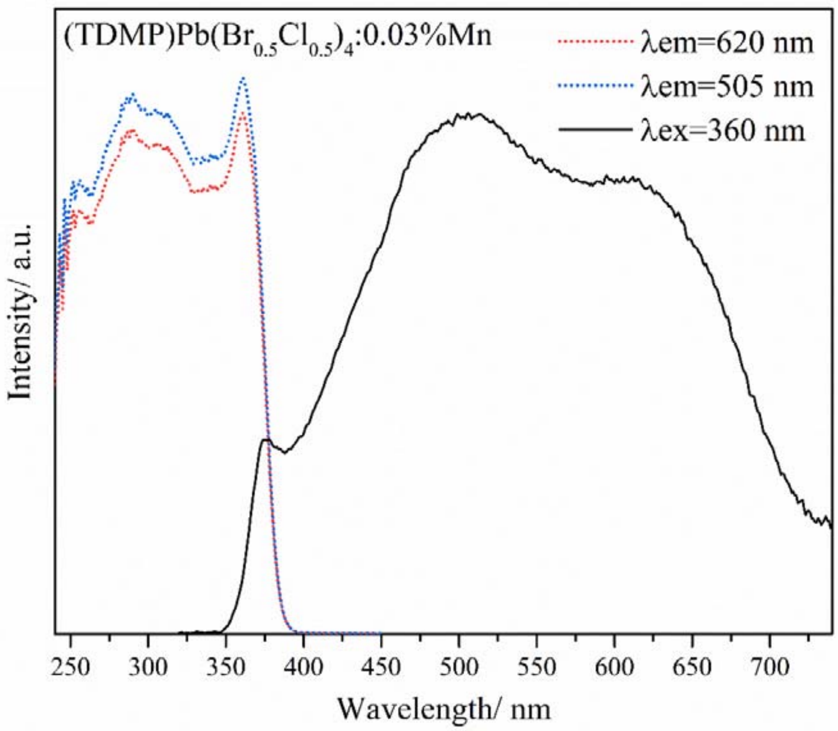

Figure S9. Photoluminescence excitation and emission spectra of $(TDMP)Pb(Br_{0.5}Cl_{0.5})_4$:0.03%Mn.

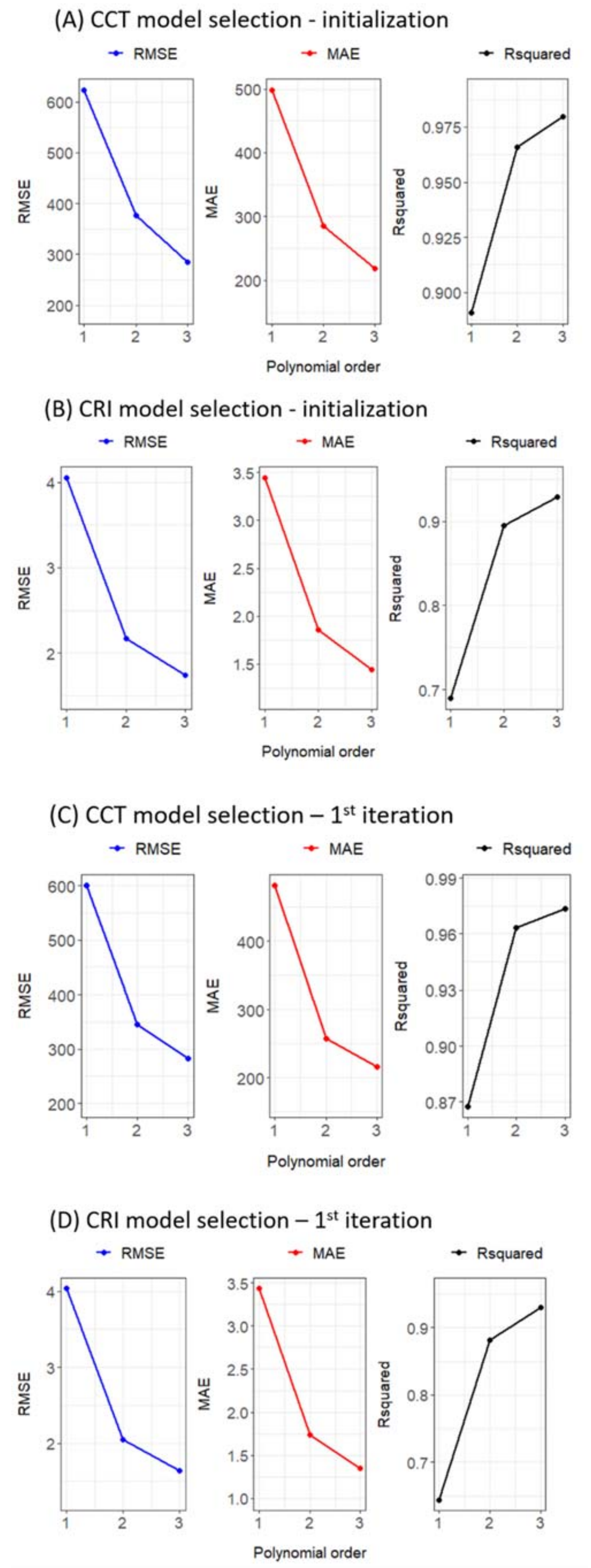

(A) CCT model selection - initialization
RMSE
MAE
Rsquared
600
500
400
300
200
500
400
300
200
0.975
0.950
0.925
0.900
1
2
3
Polynomial order
(B) CRI model selection - initialization
RMSE
MAE
Rsquared
4
3
2
3.5
3.0
2.5
2.0
1.5
0.9
0.8
0.7
1
2
3
Polynomial order
(C) CCT model selection – 1st iteration
RMSE
MAE
Rsquared
600
500
400
300
200
400
300
200
0.99
0.96
0.93
0.90
0.87
1
2
3
Polynomial order
(D) CRI model selection – 1st iteration
RMSE
MAE
Rsquared
4
3
2
3.5
3.0
2.5
2.0
1.5
1.0
0.9
0.8
0.7
1
2
3
Polynomial order

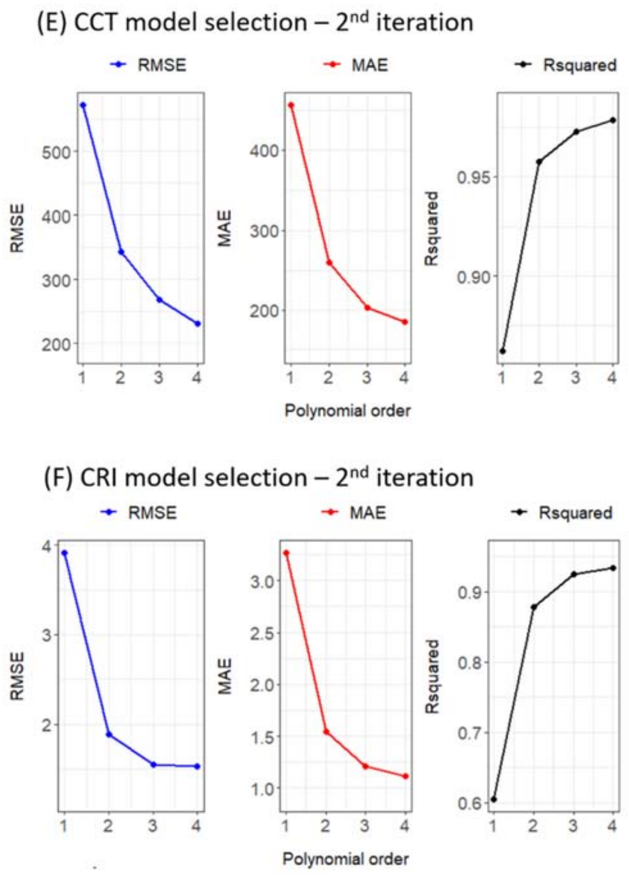

Figure S10. The RMSE (Root Mean Square Error), MAE (mean absolute error) and R-Squared of the polynomial regression models established in the iteration process.
